# Peak value of serum KL-6 may be useful for predicting poor prognosis of severe COVID-19 patients

**DOI:** 10.1186/s40001-022-00690-3

**Published:** 2022-05-19

**Authors:** Shuhei Maruyama, Yasushi Nakamori, Hitoshi Nakano, Keiko Tsuyumu, Shuji Kanayama, Hiromu Iwamura, Daiki Wada, Tomoyuki Yoshihara, Fukuki Saito, Kazuhisa Yoshiya, Yasuyuki Kuwagata

**Affiliations:** 1 Department of Emergency and Critical Care Medicine, Kansai Medical University Medical Center, 10-15 Fumizono-cho, Moriguchi, Osaka 570-8507 Japan; 2 Department of Emergency and Critical Care Medicine, Kansai Medical University Hospital, 2-3-1 Shinmachi, Hirakata, Osaka 573-1191 Japan

**Keywords:** KL-6, COVID-19, Prognosis, Biomarker

## Abstract

**Background:**

Serum Krebs von den Lungen 6 (KL-6), which reflects alveolar epithelial injury, was reported to be useful to predict the progression of pneumonitis induced by COVID-19 in the early phase. This study aimed to evaluate the peak value of serum KL-6 during hospitalization for COVID-19 to discover a more useful biomarker for predicting prognosis in COVID-19 patients.

**Methods:**

In this retrospective, single-center, observational study, we analyzed the data of 147 hospitalized patients who required supplemental oxygen, high-flow oxygen therapy, or invasive mechanical ventilation for respiratory failure due to COVID-19 from March 2020 to February 2021. We extracted data on patient sex, age, comorbidities, treatment, and biomarkers including the initial and peak values of KL-6. Inclusion criteria were examination of the studied biomarkers at least once within 3 days of admission, then at least once a week, and at a minimum, at least twice during the entire hospitalization. Area under the receiver operating curve (AUC) was analyzed to determine the accuracy of several biomarkers including KL-6 and LDH for predicting poor prognosis defined as survivors requiring invasive mechanical ventilation for over 28 days or non-survivors of COVID-19. Univariable and multivariate logistic regression analyses were performed to investigate the prognostic value of the baseline characteristics and biomarkers.

**Results:**

Among the 147 patients, 108 (73.5%) had a good prognosis and 39 (26.5%) had a poor prognosis. The AUC analysis indicated that peak KL-6 showed precise accuracy in the discrimination of patients with poor prognosis (AUC 0.89, *p* < 0.001). The best cut-off value for KL-6 concentration was 966 U/mL (sensitivity 81.6%, specificity84.3%). After adjustment, increasing peak values of KL-6 or LDH were associated with a high risk of poor prognosis, with an adjusted odds ratio of 1.35 for peak value of KL-6, per 100 U/mL increase (95% CI 1.17–1.57, *p* < 0.001) and 2.16 for peak value of LDH, per 100 U/L increase (95% CI 1.46–3.20, *p* < 0.001).

**Conclusions:**

Peak values of KL-6 and LDH measured during hospitalization might help to identify COVID-19 patients with respiratory failure who are at higher risk for a poor prognosis.

## Background

Severe acute respiratory syndrome coronavirus 2 (SARS-CoV-2) was first identified in December 2019 in Wuhan, China, as the cause of a respiratory illness designated coronavirus disease 2019 (COVID-19). The clinical course ranges from asymptomatic to critically ill, and approximately 5–20% of patients with COVID-19 develop severe pneumonitis, with some progressing to life-threatening respiratory failure, acute respiratory distress syndrome (ARDS), multiple organ failure, and various pathological conditions [1–5].

Krebs von den Lungen 6 (KL-6) is a high-molecular-weight mucin-like glycoprotein produced by type II pneumocytes and bronchial epithelial cells in the healthy lung [6]. Most attention has been focused on KL-6 as a diagnostic and prognostic tool in ARDS, interstitial lung diseases, hypersensitivity pneumonitis, and collagen vascular disease-associated interstitial pneumonitis [7–9]. Previous studies have shown that the expression of KL-6 protein correlates with altered alveolar capillary permeability, suggesting a link between high serum levels of KL-6 and alveolar epithelial barrier dysfunction, and the subsequent onset of ARDS [7, 10–12]. Thus, even in pneumonitis induced by COVID-19, serum KL-6 levels might be useful for determining prognosis and evaluation of therapeutic response. Several studies have reported that elevated serum level of KL-6 in patients in the early phase of COVID-19 could predict risk stratification in COVID-19 and progression to severe disease including the development of pulmonary fibrotic sequelae and death [13–16]. However, these studies involved a small number of patients.

We speculated that presence of an elevated serum level of KL-6 after hospitalization might be more meaningful than that in the early phase for the prognosis of lung injury and life expectancy when the response to treatment was not good. However, no articles have reported targets for serum KL-6 either in the early period or for the entire period of hospitalization in patients with severe COVID-19.

As well, lactate dehydrogenase (LDH), a non-specific cytotoxic marker, has been used for the evaluation of various lung diseases such as interstitial lung disease and ARDS. A previous study reported that LDH levels measured at the earliest time point in hospitalization were associated with poor prognosis even in COVID-19 patients [17].

Thus, the aim of our study was to investigate whether the peak values of serum biomarkers including KL-6 and LDH during hospitalization were useful indicators to predict poor outcome in COVID-19 patients with severe pneumonitis who either required respiratory support for over 28 days or died.

## Methods

### Study scheme

This was a retrospective, single-center, observational study. In total, 249 consecutive patients who were diagnosed as having COVID-19 confirmed by polymerase chain reaction test for SARS-CoV-2 from sputum or nasopharyngeal swab and who were hospitalized in the Department of Emergency and Critical Care Medicine, Kansai Medical University Medical Center, Osaka, Japan, between March 2020 and February 2021 were potentially eligible for the study. The inclusion criteria were the requirement of supplemental oxygen, high-flow oxygen therapy, or invasive mechanical ventilation (IMV) and to have the studied biomarkers examined at least once within 3 days of admission, then at least once a week, and at a minimum, at least twice during the entire hospitalization. In principle, KL-6 was measured once a week, but if the attending physician decided that additional tests were needed, we performed them as appropriate. The exclusion criteria were no requirement for supplemental oxygen and not meeting the above-mentioned examination frequency. After application of the exclusion criteria, 147 patients were selected. We defined the survivors who required supplemental oxygen, high-flow oxygen therapy, or IMV for less than 28 days as having a good prognosis and the survivors requiring IMV for over 28 days and the non-survivors as having a poor prognosis. Because the outcome in this study was decided at discharge and some patients were transferred to secondary care hospitals, we could not obtain a final long-term prognosis, e.g., long-term hospitalization or death after transfer. For this reason, the poor prognosis group included the survivors who required IMV for over 28 days. Clinical outcomes were monitored up to March 31, 2021. Because of the anonymous nature of the data, the requirement for informed consent was waived. Study approval was obtained from the institutional review board of Kansai Medical University Medical Center (Approval No.: 2021007).

### Data collection

We collected and analyzed physical examination, medical history, hematological, and biochemical data, treatments, and period of hospitalization as obtained from the electronic medical records of the patients with SARS-CoV-2 infection. Data collected at admission included sex, age, race, Sequential Organ Failure Assessment (SOFA) score at baseline, body mass index, smoking history, comorbidities (hypertension, diabetes mellitus, hyperlipidemia, cardiovascular disease, chronic obstructive pulmonary disease, chronic renal disease, previous cancer, autoimmune disease, and steroid use), received treatment (antiviral drugs, immunomodulatory drugs, and anticoagulant drugs), received organ support therapy (supplemental oxygen or high-flow oxygen therapy, IMV, and hemodialysis), days from onset to admission and to IMV, number of hospital days, and outcome (discharge, transfer to another hospital, and death). The value of each biomarker obtained within the first 3 days after admission was defined as the initial value, and we collected initial values for serum KL-6, LDH, D-dimer, ferritin, procalcitonin, C-reactive protein, and blood cell analysis factors (white blood cell and lymphocyte counts). The highest value of each biomarker obtained over the total hospital stay was defined as the peak value, and we collected the peak values for serum KL-6, LDH, D-dimer, and ferritin. Serum levels of KL-6, LDH, ferritin, and D-dimer were measured by latex-enhanced immunoturbidimetry using a Nanopia KL-6 reagent kit (Sekisui Medical Co., Ltd., Tokyo, Japan), IFCC method using a lactate dehydrogenase kit (FUJIFILM Wako Pure Chemical Co., Ltd., Osaka, Japan), latex-enhanced immunoturbidimetry using a FER-latex X2 SEIKEN CN kit (Denka Co., Ltd., Tokyo, Japan), and a LIASAUTO D-dimer NEO reagent kit (Sysmex Co., Ltd., Hyogo, Japan), respectively.

### Treatment protocol for COVID-19

As the basic treatment strategy, IMV was conducted for the patients with COVID-19 who required either supplemental oxygen at 5 L/min or more or high-flow oxygen therapy to maintain their SpO_2_ at a minimum of 93%. All patients were administered an antiviral drug and immunomodulator drug. A rest lung strategy of high positive end expiratory pressure and low tidal volume was performed along with prone position therapy for 16 h a day. We did not administer continuous muscle relaxants. Antiviral therapy included either remdesivir or favipiravir. We basically administered remdesivir intravenously at a 200-mg loading dose on day 1, followed by a 100-mg maintenance dose administered daily on days 2 through 5. Patients with an estimated glomerular filtration rate of < 30 mL/min were administered favipiravir orally, instead of remdesivir, at a 3600-mg loading dose on day 1, followed by a 1800-mg maintenance dose administered daily on days 2 through 10. All patients enrolled in this study received methylprednisolone and tocilizumab. Methylprednisolone was administered intravenously at 125 mg on days 1 through 3 and at 40 mg on days 4 through 10, but steroid pulse therapy (methylprednisolone intravenously at 250 or 500 mg per day for 3 days) was only administered as needed. Tocilizumab was administered intravenously at 8 mg/kg on day 1.

### Statistical analysis

To investigate the relationship between the biomarkers and outcome, we performed several analyses. We studied the relationship between the basal serum levels of the biomarkers and outcome first, that between the serum peak levels of KL-6, LDH, D-dimer, and ferritin, and outcome second, and finally, that between the serum levels of the biomarkers including KL-6 and outcome. We used the Mann–Whitney *U* test to compare the median values of the continuous variables and Fisher’s exact test to compare the proportions of categorical variables between the groups. During the period from 8 to 28 days after onset, each of the KL-6 and LDH levels that were measured in the good prognosis group and poor prognosis group were analyzed using the Mann–Whitney *U* test. Receiver operating characteristics (ROC) curves were plotted for the poor prognosis group. The accuracy of each variable was assessed by calculating the area under the curve (AUC), the best cut-off value, and the sensitivity and specificity. Additionally, to identify variables associated with the poor prognosis group, we built univariate regression models. Clinically relevant variables significantly associated with the poor prognosis group (*p* < 0.001) were entered into multivariable models. Collinearity between the variables was tested for each model. Logistic regression selection was performed with the forced entry procedure. In all tests, *p* values < 0.05 were considered to indicate statistical significance. Statistical analysis and graphic representation of the data were performed with GraphPad Prism 9.0 software and SPSS 28.0 software.

## Results

**Fig. 1:**
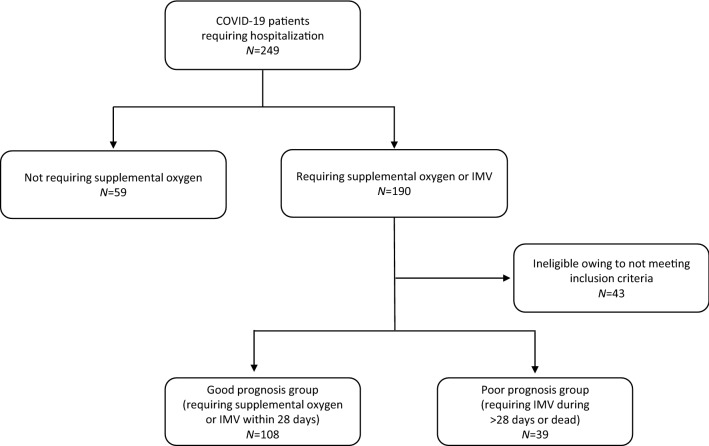
Patient flowchart of this study. *IMV* invasive mechanical ventilation

In total, 249 COVID-19 patients were hospitalized at the Department of Emergency and Critical Care Medicine, Kansai Medical University Medical Center, Osaka, Japan, between May 2020 and February 2021. After removing the patients meeting the exclusion criteria, 147 patients were included in the analyses, of whom 108 (73.4%) were classified into the good prognosis group, and 39 (26.5%) were classified into the poor prognosis group (Fig. 1). Patients in the poor prognosis group were significantly older than those in the good prognosis group (median 79 vs. 69 years, *p* < 0.001). SOFA score at admission, prevalence of diabetes mellitus, and the rates of administration of unfractionated heparin, IMV, and hemodialysis were significantly higher in the poor prognosis group than those in the good prognosis group. There was no significant difference in time of onset to admission (median 7.0 vs. 7.0 days, *p* = 0.65) and to IMV (median 8.0 vs. 8.0 days, *p* = 0.67). In the good prognosis group, there were 13 (12%), 8 (7%), and 84 patients (78%) with 2, 3, and ≥ 4 measured KL-6 values, respectively. Similarly, in the poor prognosis group, there were 2 (5%), 0 (0%), and 37 patients (95%) with these values (Table 1).

**Table 1:** Baseline characteristics, treatments during hospitalization, and outcome

Characteristics	Total *N* = 147	Good prognosis group *N* = 108	Poor prognosis group *N* = 39	*P* value
Men, *N*	105 (71%)	78 (72%)	27 (69%)	0.84
Age (years)	73 [61–79]	69 [57–77]	79 [71–84]	< 0.001
Asian, *N*	147 (100%)	108 (100%)	39 (100%)	
SOFA score at admission	5 [3–8]	4 [3–7]	7 [6–11]	< 0.001
Body mass index (kg/m^2^)	23.3 [21.3–25.7]	23.5 [21.5–26.2]	22.9 [20.3–25.3]	0.58
Smoking history, *N*				
None	48 (33%)	37 (34%)	11 (28%)	
Past	65 (44%)	44 (41%)	21 (54%)	
Current	12 (8.2%)	11 (10%)	1 (2.6%)	0.18
Comorbidities, *N*				
Hypertension	83 (57%)	61 (57%)	22 (56%)	> 0.99
Diabetes mellitus	58 (40%)	37 (34%)	21 (54%)	0.037
Hyperlipidemia	49 (33%)	33 (31%)	16 (41%)	0.24
Cardiovascular disease	30 (20%)	20 (19%)	10 (26%)	0.36
COPD	17 (12%)	10 (9.3%)	7 (18%)	0.15
CKD	30 (20%)	18 (17%)	12 (31%)	0.068
Previous cancer	20 (14%)	14 (13%)	6 (15%)	0.79
Autoimmune disease	9 (6.1%)	6 (5.6%)	3 (7.7%)	0.70
Steroid user	5 (3.4%)	2 (1.9%)	3 (7.7%)	0.12
Treatment, *N*				
Favipiravir	70 (51%)	48 (44%)	22 (56%)	0.26
Remdesivir	88 (60%)	66 (61%)	22 (56%)	0.70
Glucocorticoids	141 (96%)	103 (95%)	38 (97%)	> 0.99
Tocilizumab	115 (78%)	82 (76%)	33 (85%)	0.37
Nafamostat	114 (78%)	81 (75%)	33 (85%)	0.27
Unfractionated heparin	115 (78%)	80 (74%)	35 (90%)	0.044
Organ support therapy				
Supplemental oxygen or high-flow therapy	39 (28%)	41 (38%)	0 (0%)	< 0.001
IMV	105 (71%)	67 (62%)	38 (97%)	< 0.001
Hemodialysis	30 (20%)	11 (10%)	19 (49%)	< 0.001
Time (days)				
Onset to admission	7 [4–9]	7 [4–9]	7 [3–9]	0.65
Onset to IMV	8 [6–10]	8 [6–10]	8 [6–10]	0.67
Hospital day	16 [9–27]	14 [8–22]	25 [15–35]	< 0.001
Outcome, *N*				
Discharge	27 (18%)	27 (25%)	0 (0%)	
Transfer to hospital	86 (59%)	79 (73%)	7 (18%)	
Death	34 (23%)	2 (2%)	32 (82%)	
Number of KL-6 values				
2	15 (10%)	13 (12%)	2 (5%)	
3	8 (5%)	8 (7%)	0 (0%)	
≥ 4	121 (82%)	84 (78%)	37 (95%)	
Median	13	10	20	

Good prognosis group included patients requiring oxygen or IMV within 28 days. Poor prognosis group included patients requiring IMV over 28 days or who were discharged to death

Data are expressed as *N* (%) or median (1st IQR-3rd IQR). *p* values were calculated with the Mann–Whitney *U* test or Fisher test as appropriate

*SOFA* sequential organ failure assessment, *COPD* chronic obstructive pulmonary disease, *CKD* chronic kidney disease, *IMV* invasive mechanical ventilation, *KL-6* Krebs von den Lungen-6

The initial and peak values of serum KL-6 were significantly higher in the poor prognosis group than those in the good prognosis group (median 477 vs. 317 U/mL, *p* < 0.001; median 1472 vs. 497 U/mL, *p* < 0.001, respectively). The other values that were significantly higher in the poor prognosis group than good prognosis group were the initial and peak values levels of serum LDH (*p* = 0.009; *p* < 0.001, respectively) and D-dimer (*p* = 0.004; *p* < 0.001, respectively) and the peak values levels of ferritin (*p* = 0.021) (Table 2).

**Table 2:** Laboratory findings of COVID-19 patients in the good and poor prognosis groups

	Total *N* = 147	Good prognosis *N* = 108	Poor prognosis *N* = 39	*p* value
Initial value				
KL-6, U/mL	356 [238–539]	317 [227–473]	477 [304–712]	< 0.001
LDH, U/L	359 [293–495]	349 [267–465]	453 [318–583]	0.009
D-dimer, ng/mL	1.5 [0.7–3.3]	1.3 [0.5–3.0]	2.7 [1.4–5.5]	0.004
Ferritin, ng/mL	666 [424–1283]	707 [425–1219]	610 [422–1589]	0.82
PCT, ng/mL	0.18 [0.09–0.41]	0.16 [0.08–0.38]	0.22 [0.13–0.58]	0.13
CRP, mg/dL	8.4 [4.7–15.6]	9.2 [5.1–15.5]	6.8 [2.9–17.5]	0.20
WBC, /μL	7700 [5200–10700]	7700 [5300–10400]	7700 [4300–12200]	0.69
Lymphocyte, /μL	683 [375–964]	673 [380–948]	684 [362–998]	0.98
Peak value				
KL-6, U/mL	709 [406–1165]	497 [355–826]	1472 [1137–2139]	< 0.001
LDH, U/L	493 [345–668]	415 [328–533]	755 [628–1155]	< 0.001
D-dimer, ng/mL	9.8 [3.8–30.6]	6.8 [2.8–20.2]	30.7 [11.5–44.3]	< 0.001
Ferritin, ng/mL	911 [536–1580]	862 [475–1428]	1147 [635–2104]	0.021

Good prognosis group included patients requiring supplemental oxygen or invasive mechanical ventilation within 28 days. Poor prognosis group included patients requiring invasive ventilation over 28 days or who were discharged to death

Initial value was defined the first value obtained within the 3rd day of hospitalization; peak value was defined the peak value during the entire hospitalization

Data are expressed as median (1st IQR-3rd IQR). *p* Values were calculated with the Mann–Whitney *U* test

*KL-6* Krebs von den Lungen 6, *LDH* lactate dehydrogenase, *PCT* procalcitonin, *CRP* C-reactive protein, *WBC* white blood cell count

**Fig. 2:**
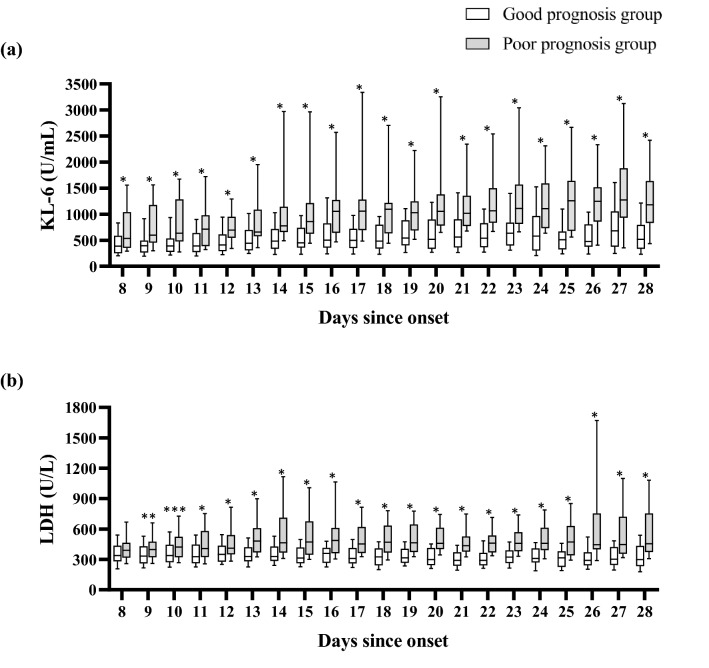
Kinetics of the KL-6 (**a**) and LDH (**b**) levels in the good prognosis group (*n* = 108) and poor prognosis group (*n* = 39). Boxes indicate the median (interquartile range) and whiskers the 10th and 90th percentiles. *p < 0.01, **p = 0.034, ***p = 0.022 by Mann–Whitney *U* test. Significantly higher levels of KL-6 and LDH at all points except for LDH on day 8 were observed in the poor prognosis group. *KL-6* Krebs von den Lungen-6, *LDH* lactate dehydrogenase

**Fig. 3:**
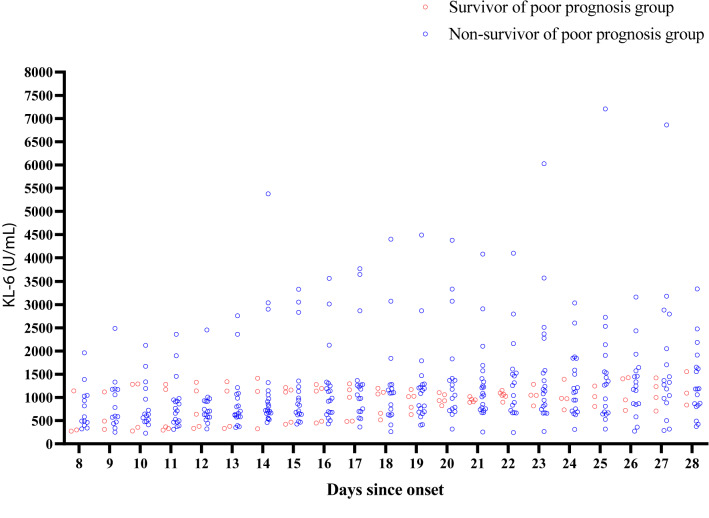
KL-6 levels of survivors and non-survivors in the poor prognosis group

The values of KL-6 and LDH measured on each of days 8 through 28 after onset in the poor prognosis group were significantly higher than those in the good prognosis group (*p* < 0.01), except for that of LDH on day 8 (*p* = 0.18) (Fig. 2). KL-6 values of the survivors and non-survivors in the poor prognosis group are presented in Fig. 3.

**Fig. 4:**
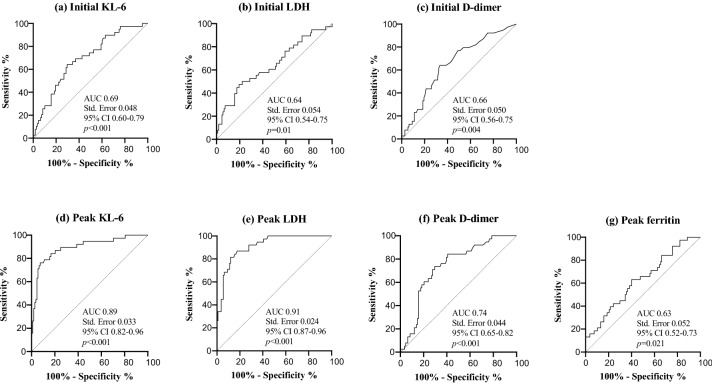
Receiver operating characteristic curve analyses of initial and peak values of serum biomarkers for predicting poor prognosis, defined as requiring IMV over 28 days or death in COVID-19 patients requiring supplemental oxygen or IMV. Initial value was defined as the first value obtained within the 3rd day of hospitalization, and peak value was defined as the highest value obtained during the entire hospitalization. Among the initial values, the optimal cut-off values of KL-6 (**a**), LDH (**b**), and D-dimer (**c**) were 412 U/mL (sensitivity 64.1%, specificity 70.4%), 408 U/L (55.3%, 65.7%), and 1.85 ng/mL (64.1%, 66.7%), respectively. Among the peak values, the optimal cut-off values of KL-6 (**d**), LDH (**e**), D-dimer (**f**), and ferritin (**g**) were 966 U/mL (81.6%, 84.3%), 556 U/L (86.8%, 82.4%), 16.2 ng/mL (71.1%, 72.2%), and 1152 U/L (50.0%, 65.4%), respectively. *KL-6* Krebs von den Lungen-6, *LDH* lactate dehydrogenase, *AUC* area under the curve, *IMV* invasive mechanical ventilation

Area under the ROC curve analysis indicated that the initial value of KL-6 showed good accuracy in discriminating the patients with poor prognosis (AUC 0.69, std. error [SE] 0.048, 95% confidence interval [CI] 0.60–0.79, *p* < 0.001). The best cut-off value for the KL-6 concentration was 412 U/mL (sensitivity 64.1%, specificity 70.4%). In the analysis of peak values, KL-6 and LDH showed precise accuracy in discriminating the patients with poor prognosis (AUC 0.89, SE 0.033, 95% CI 0.83–0.96, *p* < 0.001 and AUC 0.91, SE 0.024, 95% CI 0.87–0.96, *p* < 0.001, respectively). The best cut-off values for KL-6 and LDH concentrations were 966 U/mL (sensitivity 81.6%, specificity 84.3%) and 556 U/mL (sensitivity 86.8%, specificity 82.4%), respectively (Fig. 4). After adjustment, increasing peak values of KL-6 and LDH were associated with a high risk of poor prognosis, with an adjusted odds ratio of 1.35 for peak value of KL-6, per 100 U/mL increase (95% CI 1.17–1.57, *p* < 0.001), and 2.16 for peak value of LDH, per 100 U/L increase (95% CI 1.46–3.20, *p* < 0.001) (Table 3).

**Table 3:** Analysis of predictors in the poor prognosis group using multivariable logistic regression

	Odds ratio (95% CI)	*p* value
Age (years)	1.14 [1.05–1.23]	0.002
Peak value of D-dimer, per ng/mL increase	1.00 [0.99–1.01]	0.783
Peak value of KL-6, per 100 U/mL increase	1.35 [1.17–1.57]	< 0.001
Peak value of LDH, per 100 U/L increase	2.16 [1.46–3.20]	< 0.001

*CI* confidence interval, *KL-6* Krebs von den Lungen 6, *LDH* lactate dehydrogenase

## Discussion

This study investigated the relationship between the levels of biomarkers including KL-6 in the early phase and peak serum values during the overall hospitalization and poor prognosis defined as prolonged IMV for over 4 weeks or discharge to death in patients requiring respiratory support due to respiratory failure induced by COVID-19 pneumonitis. Both the initial and peak serum values of KL-6 and LDH were significantly higher in the poor prognosis group than those in the good prognosis group. In addition, levels of peak KL-6 higher than 966 U/mL and peak LDH higher than 556 U/L were shown to be precise prognostic factors for predicting prolonged IMV and discharge to death.

Two patients in the good prognosis group had peak KL-6 and LDH values of 808 U/mL and 488 U/L and 1172 U/mL and 549 U/L, respectively. These patients were very old and died due to exacerbation of underlying diseases during hospitalization. The seven patients in the poor prognosis group who survived tended to have comparatively lower KL-6 levels in the group from the 8th to 28th day after onset. Although statistical analysis was not possible due to the small sample size, the higher KL-6 levels in the patients in the poor prognosis group appeared to be related to their death.

A previous study showed that the value of serum KL-6 at the time of enrollment was a precise predicter associated with death using a cut-off value for KL-6 of > 1000 U/mL (AUC 0.85) [15]. Our study found a cutoff value of KL-6 in the initial phase (i.e., the initial measurement within the first 3 days of hospitalization) that was lower (412 U/mL), but the accuracy was not so high (AUC 0.69) compared with that of the previous report. It might be difficult to determine an initial value of KL-6 in a consistent phase for each patient because hospitalization and enrollment in our study differed from those of other previous studies as the period from the onset of COVID-19 varied.

A previous retrospective longitudinal study reported a delayed peak day in severely ill patients compared to non-severe patients (21.8 ± 6.0 vs. 15.3 ± 7.0 days, *p* = 0.015), indicating that it is likely that the peak value of KL-6 will be missed if only early-phase values are evaluated [16]. Our study assessed the change in KL-6 values from onset to 28 days, with the timing of the peak KL-6 value occurring in the fourth week within the survey period. After the fifth week, some patients were transferred to other hospitals or had died; therefore, we presented changes in the serial values only within the first 4 weeks after onset.

The elevation of KL-6 in the severely ill patients was correlated with the activation of immunity. Generally, steroids are administered to treat the “cytokine storm” caused by COVID-19, and lung injury due to an excessive immune response as indicated by the peak value of KL-6 might inform clinicians of the necessity to change the therapeutic strategy such as by administering additional immunosuppressants.

The high-molecular-weight glycoprotein KL-6 is predominantly found on alveolar type II cells in the normal lung [6, 11, 18]. Lung compartment KL-6 is produced and released by proliferating alveolar type II cells following injury to alveolar type I cells. Because an increase in spillover toward the systemic circulation is caused by leakage resulting from the damaged integrity of the alveolo-capillary membrane, the KL-6 level must reflect the severity of alveolar epithelial injury [9, 19–22]**.** Serum KL-6 levels correlate with indices of alveolar capillary permeability, and serum KL-6 is elevated in various respiratory diseases including interstitial lung disease and ARDS, regardless of the etiology [6, 7, 9, 11, 18]. KL-6 promotes chemotaxis of human fibroblasts and was found to accelerate the proliferation of lung fibroblasts and inhibit the apoptosis of all human lung fibroblasts examined [23, 24]. Inappropriate ventilator settings for ARDS cause ventilator-induced lung injury and raise serum KL-6 as a result of alveolar epithelial damage. Thus, if serum KL-6 remains elevated over time, the patient’s breathing pattern and ventilator settings should be confirmed to be appropriate for the rest lung strategy used [25]. It stands to reason that efforts to limit the rise of peak KL-6 might help to suppress lung injury. Because elevated serum KL-6 indicates a prognosis that reflects injury to the alveolar epithelium and the development of pulmonary fibrotic sequelae [14], changes in the KL-6 value should be monitored throughout the treatment period.

In addition to peak KL-6, our study found the peak level of serum LDH to also be a precise prognostic marker. Multiple organs other than the lungs are frequently affected in severely ill COVID-19 patients. Therefore, an elevated LDH level reflecting non-specific tissue injury (e.g., hemolysis, liver injury, myocardial infarction, lung injury, or rhabdomyolysis) might be an important biomarker in addition to KL-6.

Compared to the findings of several previous reports, the initial value of serum KL-6 in the present study was not a useful biomarker for predicting poor prognosis in COVID-19 patients. Serial measurement of serum KL-6 might inform clinicians of treatment refractoriness and poor prognosis. If serum KL-6 remains elevated over time, the treatment strategy might need to be changed such as by administering immunomodulators and adjusting respiratory settings to prevent ventilator-induced lung injury.

This study has several limitations. First, it was a retrospective observational study, and 43 patients were excluded due to missing data and not meeting the inclusion criteria. Second, the number of patients might not be sufficient to perform a valid statistical analysis, even though the number of samples was larger than those of the previous reports [13–16]. Further research with an increased sample size should be performed in the future. Third, some of the patients were transferred to secondary hospitals after their general condition had stabilized, and it was thus not possible to evaluate the entire period of COVID-19 therapy, especially in the patients with a milder form of COVID-19. The peak values reported in this study might not be the true values occurring throughout the entire COVID-19 disease period because some of the patients were transferred to other hospitals after their general condition had stabilized. Fourth, the initial values should be correlated with disease onset, and therefore, the initial values defined in this study may not necessarily be the true initial values. Fifth, kinetic comparisons of the KL-6 and LDH values in the two group were not evaluated daily.

## Conclusion

To our knowledge, the present study was the first to focus on the peak value of KL-6 during the entire hospitalization of patients with COVID-19. The peak values of KL-6 and LDH during hospitalization might be more useful than the initial and peak value of other biomarkers to predict poor outcome defined as prolonged use of IMV and discharge to death.

## Data Availability

The datasets analyzed during the current study are available from the corresponding author on reasonable request.

## Abbreviations

ARDS: Acute respiratory distress syndrome
AUC: Area under the curve
CI: Confidence interval
COVID-19: Coronavirus disease 2019
IMV: Invasive mechanical ventilation
KL-6: Krebs von den Lungen 6
LDH: Lactate dehydrogenase
ROC: Receiver operating characteristics
SARS-CoV-2: Severe acute respiratory syndrome coronavirus 2
